# Efficacy of Warmup Using a Flexible Bar to Increase Countermovement Jump

**DOI:** 10.3390/sports12110298

**Published:** 2024-11-01

**Authors:** Benjamin J. Snyder, Anthony Caterisano, Evan P. DiMaggio, Jackson D. King

**Affiliations:** Department of Health Sciences, Furman University, Greenville, SC 29613, USA; tony.caterisano@furman.edu (A.C.); evan.dimaggio@furman.edu (E.P.D.)

**Keywords:** American football, electromyography, postactivation performance enhancement, jump height, Tsunami bar

## Abstract

Preparation of athletes to begin a competition or enter mid-competition with the ability to reach peak performance immediately may be partly dependent on the efficacy of the warmup protocol. Previous research on flexible barbells reported significant differences in muscle activation when compared to steel barbells. The manufacturers of handheld flexible bars with a combined weight of 3.63 kg claim that using them immediately before competition helps increase power and jump height. The purpose of the study was to determine the effects of maximal jumping with handheld flexible bars on maximal jump parameters in Division-I American collegiate football players. Participants completed 10 warmup jumps with no weight, flexible barbells, or similarly weighted dumbbells and immediately completed a maximal countermovement jump. When comparing the effects of different high-velocity warmup (HVW) protocols, there were no differences in any jump parameters as measured by a portable force plate. Likewise, the use of flexible bars during the warmup did not result in enhanced muscle activity when executing a maximal jump in comparison to control conditions. Based on these results there is no evidence to support the use of flexible bars during a warmup just prior to entry into a competition to enhance jumping power.

## 1. Introduction

Athletes and coaches are continually searching for the best pregame routines to achieve peak performance. In practice, dynamic warmups have largely replaced static stretching before competition, and recent reviews have concluded that in most situations and for most populations, static stretching can be deleterious to performance of power activities, while dynamic warmups may enhance performance and are very unlikely to decrease performance of dynamic and high-velocity activities [1,2]. Additionally, a number of routines have been proposed to enhance power through postactivation performance enhancement (PAPE) [3,4] of lower body power. However, many of those involve the use of barbells at heavy loads and effects, if present, were often not seen for several minutes following the conditioning contraction [5,6,7,8]. Patti et al. explored the effect of sprint warmups on a series of countermovement jumps and found that the optimal sprint recovery time for CMJ performance was 330 s [9].

Recently, handheld flexible bars have found limited usage in collegiate and high school training regimens, purportedly to enhance power production and jump performance when used immediately prior to substituting into a game. Research into the efficacy of a flexible barbell produced by the same manufacturer revealed that performing squats with a flexible barbell loaded with only 30% 1RM results in significantly greater leg and trunk muscle activity and higher ground reaction forces than squats with a standard Olympic barbell, possibly due to the momentum-generated oscillation of the bar at higher speeds [10]. A more recent study confirmed that peak ground reaction force during a squat is higher than an Olympic barbell when performed at a 90 reps per minute cadence [11]. Subsequent research from the same laboratory compared training with a flexible barbell (“Tsunami bar”) to a combined weight training protocol of speed-lifts and plyometric exercises and found that while both increased power significantly, training with the flexible barbell resulted in superior increases in the Margaria–Kalamen stair test and the long jump [12]. Unpublished pilot data from our lab indicated that average force and reactive strength index were higher during a maximal set of five rebound jumps with the handheld flexible bars when compared to control conditions and holding a similarly weighted set of dumbbells. Based on these results, it is possible that the use of smaller, lighter, handheld flexible bars could also result in increased power production in a subsequent effort like a maximal jump, as claimed by the manufacturer (personal communication), but this has not been investigated. One study [13] showed that dynamic warmups with a 10% body-weighted vest results in higher long jump and vertical jump performance after a 2 min rest compared with static stretching, but only long jump was higher compared to warmups without the vest. Another study [14] measuring CMJ after a comprehensive warmup and either free-weight or band resistance showed immediate (30 s) and lasting (up to 12 min) increases in jump height, EMG activity, and other kinetic and kinematic variables, but only after band resistance. This demonstrates that the type of resistance is important in affecting subsequent dynamic performance tasks.

The manufacturer of the implement claims that a protocol of five submaximal followed by five maximal jumps while holding the flexible bars enhances subsequent power production and advocates their use by athletes just prior to their entry into the game. Therefore, the primary purpose of the present study was to compare the immediate effects of a low-weight, low-volume, high-velocity warmup protocol using the flexible bars with an identical protocol using similarly weighted dumbbells or no weight on countermovement jump force parameters and electromyographic activity. The secondary purpose was to compare electromyographic activity during the high-velocity warmup protocol itself between the flexible bars and similarly weighted dumbbells.

## 2. Materials and Methods

### 2.1. Experimental Approach to the Problem

Previous research has supported the superiority of a flexible barbell compared to a standard barbell in activating muscles during a squat and greater gains during high-power tests when training with a flexible bar [10,12]. A derivation of this product sees the user hold a smaller and lighter set of flexible bars in their hands while jumping (Flexi-Stix, LLC, Anderson, SC, USA). The manufacturer claims (personal communication) that jumping maximally with the flexible bar can improve power production during immediately subsequent efforts without the flexible bar. We therefore designed a protocol to determine if the use of a handheld flexible bar immediately (within approximately 10 s) before a maximal jump would result in greater changes to jump characteristics such as jump height, force production, and time-based variables as measured by a portable force platform or changes to leg muscle activity during a maximal jump on the force platform when compared to the use of a similarly weighted dumbbell or no weight. Each participant therefore completed a high-velocity warmup (HVW) protocol with (1) control conditions (no weight in the hands (CON)), (2) with a flexible bar in each hand (FB), or (3) with an equally weighted dumbbell in each hand (DB), after which jump variables and EMG activity during a maximal jump were compared among the three conditions. Additionally, in a separate experiment performed after the first experiment was completed, we sought to understand whether the oscillations of the flexible bar during jumping would affect muscle activity during the HVW protocol. In this experiment, we measured EMG activity of four leg muscles during five maximal jumps with either the flexible bar or the equally weighted dumbbells, with a 3 min rest in between sets. All tests were performed in one day and the order of the HVW randomized, with sufficient rest time between conditions to mitigate the effects of fatigue. It is important to note that rather than seek a way to optimize performance with the flexible bar, our goal was to test the effects of the implement using the manufacturer’s recommended routine for a game situation. Intensity, number of repetitions, and postroutine rest times were selected under this rubric.

### 2.2. Participants

Thirteen healthy athletes aged 18–21 years (mean 21.2 ± 1.1 SD) with a mean standing height of 1.85 m (±0.034 SD) and mean body weight of 98.2 kg (±7.4 SD) from a Division-I American football program were recruited directly to participate in the study during the summer offseason. In order to maintain as homogenous a population as possible, all recruits were categorized as tight ends, linebackers, or fullbacks. All had previously used the handheld flexible bars in past workout sessions and understood how to maximize the oscillations of the instrument. All participants were engaged in normal offseason training, and no recently or currently injured players were recruited to participate. Participants signed a consent form outlining the measurements to be taken and the risk associated with each. The study was reviewed and approved by the University’s Institutional Review Board following the principles of the Declaration of Helsinki prior to the initial recruitment of participants.

### 2.3. Procedures

**Figure 1:**
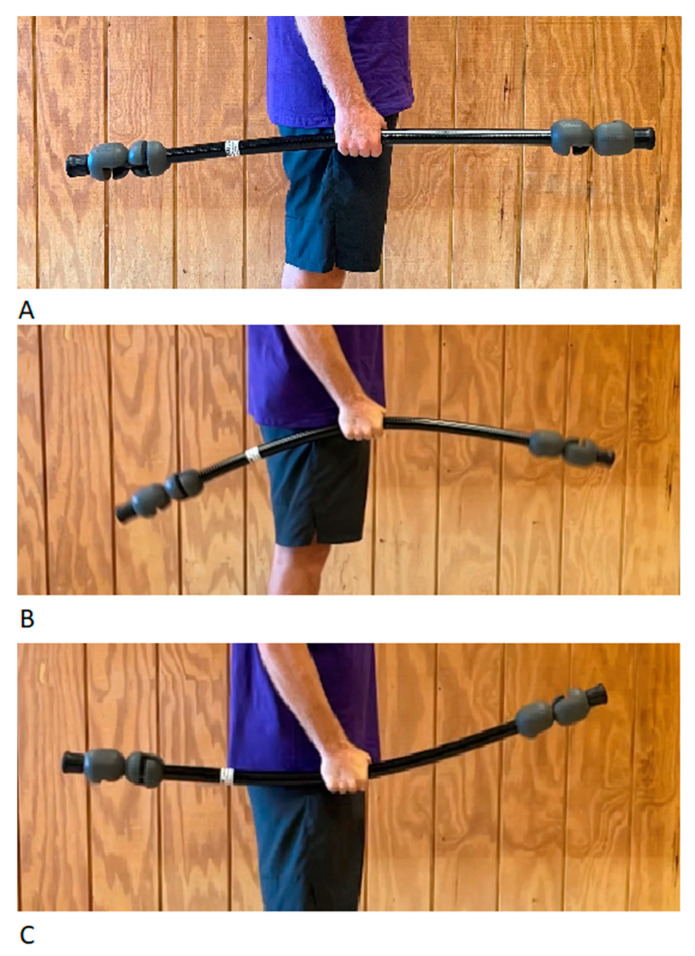
(**A**) Resting position (**B**) downward and (**C**) upward oscillatory bending of the bar during jumping. Source: authors.

Participants first reported to the athletic weight room for a familiarization session. At this time, the proper position for maximum voluntary isometric contractions (MVIC) was determined and participants instructed on how to execute a maximal contraction in each position. EMG activity for MVIC was later used to normalize maximal EMG readings during analysis. Next, the ability to properly execute jumps with the flexible bar was determined. Although they are manufactured in different lengths and weights, the flexible bars used for this experiment were 122 cm long and weighed 3.63 kg combined. Proper execution of the jump included holding the flexible bar with a neutral grip and keeping the arms straight and at their side. In this position, the flexible bar oscillated 3–4 times upward (Figure 1B) and downward (Figure 1C) from the momentum generated with every jump, and participants practiced this until they could consistently elicit this oscillation pattern. Participants were also familiarized with the procedure for measuring maximal countermovement jump (CMJ) characteristics with the portable force platform (Hawkin Dynamics, Westbrook, ME, USA). The athletes were told to keep their hands on their hips, squat to their desired depth, and then “explode off the platform” with as much force as possible.

**Figure 2:**
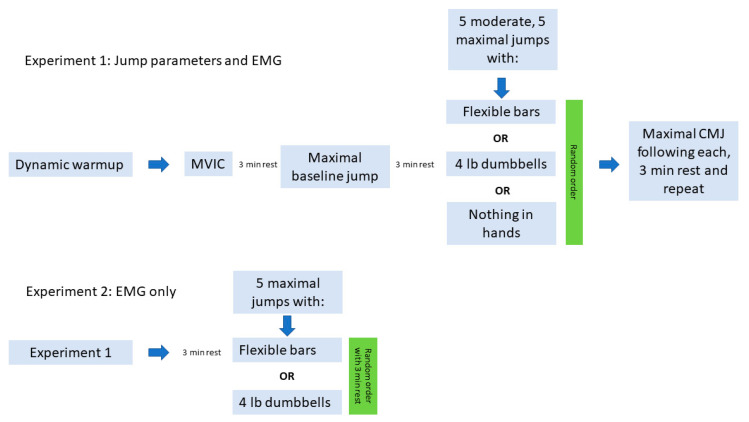
Conceptual map of project.

A minimum of 48 h later, participants reported to the weight room for the second and final testing session, which is represented in Figure 2. EMG sites for lateral gastrocnemius (LG), biceps femoris (BF), vastus lateralis (VL), and vastus medialis oblique (VMO) were first shaven, then rubbed vigorously with an alcohol pad before wireless sensors (Trigno Avanti, Delsys, Natick, MA, USA) were affixed with double-sided adhesive strips according to the SENIAM protocol [15]. To avoid excessive movement and/or detachment of the electrodes during maximal jumping, light foam prewrap was wrapped around the leg at the sensor site. Signal strength and quality were then confirmed with the EMGWorks Acquisition software v4.8.0 (Delsys, Natick, MA, USA).

The experimental protocol can be seen in Figure 2. The dynamic warmup imitated the type of routine that is used prior to participation in an American football game, and included A-skips, lateral bounds for height, straight-leg kick with skip, and dynamic marches. Participants then engaged in an MVIC by pushing as hard as possible for 3 s in different positions while attached to an immovable steel structure by a leather belt with a chain. A review of EMG normalization methods reports that the use of EMG activity during maximal isometric contractions at an arbitrary joint angle produces inter-individual reliability that is at least as high as with other methods [16]. For the VL, participants were standing in a half-squat position with 110 degrees of flexion and attempted to stand up fully by extending their knees and hips. For the VMO, participants were more upright beginning with 20 degrees of knee flexion and attempted to stand up fully by extending their knee and hips. For the LG, participants stood fully upright only had a slight bend of their knee with their ankle at 90 degrees, and they attempted to maximally plantarflex. For the BF, participants were prone on the floor with their arms at their side. While one investigator held their ankles, participants attempted to lift their torso and thighs off the floor by flexing their knees. If any movement was achieved, a second investigator put light pressure on their upper back to ensure the attempt was isometric and maximal. After the MVIC, participants rested in a seated position for 3 min (which pilot data showed was adequate for full recovery in this population). Following the rest period, subjects completed 3 maximal CMJs to determine their baseline jump parameters, then began another 3 min rest period before beginning experiment 1.

For experiment 1, participants completed a HVW protocol consisting of 5 moderate-intensity jumps and 5 maximal-intensity jumps with no pauses. During this protocol, participants held in their hands, in random order, either the flexible bar (FB), an equally weighted dumbbell set (DB), or nothing (CON). Immediately after completing the 10 jumps, participants dropped the dumbbell or flexible bar, proceeded to the force plate, and completed three maximal CMJs with 10 s between attempts, with a total of approximately 30 s elapsed from the end of the HVW protocol. This time frame was based on recommendations from the manufacturer of the flexible bar. EMG data were not collected during the HVW but during each CMJ. If the participant lost their balance, landed with part of their foot off the platform, or did not appear to jump maximally, an additional jump was completed to replace the errant one. Force plate data were transmitted via Bluetooth to a handheld tablet (Samsung, Seoul, Republic of Korea) and uploaded automatically to a cloud data site for later analysis. Participants rested in a seated position for three minutes between each warmup/CMJ cycle. Experiment 2 compared EMG activity between jumping with dumbbells vs. the flexible bar. After a 3 min rest after experiment 1 to eliminate the possibility of metabolic fatigue, participants completed five jumps with either the dumbbells or the flexible bar, in random order, separated by a 3 min rest. EMG activity of the same four muscles was collected for later analysis.

### 2.4. Data and Statistical Analysis

Analyzed jump parameters included jump height (m), average relative propulsive force (%), peak relative propulsive force (%), and time to takeoff (s). These were defined by the force plate manufacturer as follows:

*Jump height:* The change in system center of mass position between the instant of takeoff and peak positive vertical displacement of the system center of mass, calculated using the vertical velocity of the system center of mass at the instant of takeoff and the equations of uniformly accelerated motion.
*Average relative propulsive force:* The average vertical ground reaction force applied to the system center of mass during the propulsion phase as a percentage of system (body) weight.
*Peak relative propulsive force:* The peak instantaneous vertical ground reaction force applied to the system center of mass during the propulsion phase as a percentage of system (body) weight.
*Time to takeoff:* The total time taken from the initiation of movement to the instant of takeoff.

EMG signals were analyzed using the Delsys EMGWorks Analysis software v4, having been sampled at 1259 Hz using a bandwidth of 20–450 Hz, then filtered via root mean square (RMS) using a 0.125 s moving window length with a 0.0625 s overlap. Filtered signals were normalized by dividing by the MVIC RMS EMG and expressed as a percentage of the max normalized RMS (%nRMS), with the peak value during the concentric phase of the jump reported. For experiment 2, the mean of the peak %nRMS of the second through fourth jumps was reported, the first and fifth being transitional and not valid for maximal effort.

Intraclass correlation coefficients for all jump metrics were calculated with SPSS (IBM SPSS, Version 28, Armonk, NY, USA) using a two-way mixed model and were all above 0.8. For experiment 1, repeated measures ANOVA was used to test for differences (*p* ≤ 0.05) in jump characteristics and EMG activity between CON, DB, and FB. For experiment 2, a paired samples *t*-test was used to compare mean EMG activity during jumps 2–4 out of 5 with either DB or FB, with significance level set to *p* ≤ 0.05. For significant effects, Cohen’s effect sizes (*d*) were calculated, and defined as small (*d* < 0.2–0.5), medium *d* ≥ 0.5, but <0.8), large (*d* ≥ 0.8, but <1.2), or very large (≥1.2) [17]. Observed power for repeated measures based on the sample size of 13 was 30–35% for various measures.

## 3. Results

When comparing the effects of different HVW protocols, there were no differences in any jump characteristics (Table 1: experiment 1 results). Maximal jumping EMG activity of the VL following the DB HVW protocol was significantly lower than for the CON protocol, but with a very small to small effect size (*d* = 0.17) (Table 2). No other muscles were different between different HVW protocols. Table 3 (experiment 2 results) shows the comparisons of muscle activity during five maximal jumps with either the dumbbell or flexible bar. No differences in muscle activity were detected in this comparison.

## 4. Discussion

The use of a flexible barbell has previously been shown to activate leg and trunk muscles more than a standard Olympic bar [10,18] during a squat, with follow-up research confirming that training with a flexible barbell can enhance power production during high-speed movements [12]. Similar research using weights suspended from a flexible bar with elastic bands during the bench press found that this setup produced a more unstable bar path [19] (as intended) and resulted in less muscle activity in the prime movers, but more activity in the biceps brachii, acting as a stabilizer muscle [19,20].

The new product—a set of handheld flexible bars of various weights and lengths—was created with the intent of increasing muscle power in a warmup protocol to be performed with the implement just prior to entry into a competition (private communication from manufacturer). Following the manufacturer’s instructions of a set of maximal jumps with a bar in each hand, our data show that a brief bout of maximal jumping with a set of 122 cm long, 1.81 kg bars (total weight 3.63 kg) did not result in greater jump height, force parameters, or leg muscle activity when compared to a warmup with similarly weighted dumbbells or control conditions with no weight in the hands.

It is possible that the lack of an effect could be because the amount of weight used was small (representing an average of 3.7% of body weight) and that any increase in ground reaction forces due to the momentum generated by the flexible bar would not be sizable, although this was not specifically measured. One other study measured the immediate effects of unweighted jumping on explosive performance and found that it enhanced countermovement jump performance, but no more than protocols involving running, stretching, or combined running/stretching/jumping [21]. This is somewhat similar to our findings, but a direct comparison is difficult due to the inclusion of only one jumping protocol in that study. Villareal et al. [22] showed that various types of warmups, including optimally weighted jumping and low-rep squats of 80–95% 1RM, did increase jump performance more than a sport-specific warmup, but none was superior to the others. This study was different than ours, however, in that CMJ testing was performed 5 min after the warmup, whereas our CMJ testing was performed immediately after the HPW protocol. Unpublished pilot data from our own lab had indicated that a warmup that included the flexible bars may create a slightly more intense stimulus, with a higher reactive strength index and average force during a five-jump series, in comparison to no weight or similarly weighted dumbbells. While this may be an interesting avenue of research from a chronic training perspective, it appears that the additional stimulus created while using the flexible bars does not translate acutely into higher power in subsequent jumping activity. Additionally, there were no differences in EMG activity while jumping following the three HVW conditions (Table 2), and muscles were not activated differently when jumping with flexible bars vs. comparably weighted dumbbells (Table 3), confirmation that handheld flexible bars of this length and weight do not affect muscle involvement. In comparison, previous research using a flexible barbell utilized much larger weights of 47 kg [10], or 60% 1RM [18,19,20], finding alterations in muscle activity and ground reaction force, although in the latter two studies, this was mostly in stabilizer muscles rather than prime movers.

### Study Limitations

There were limitations to the study. In order to keep the group as homogenous as possible, only certain positional players were recruited, limiting the testing population and predictive power of the study. This limits the generalizability to other positions and sports. In addition, due to time restrictions, the number of muscles tested was limited to four, and not all muscles involved in the CMJ were observed.

## 5. Conclusions

In a homogenous group of Division-I American collegiate football players, five submaximal and five maximal jumps with body weight, dumbbells, or a handheld flexible bar did not result in any enhancement to jump height, jump force parameters, or lower body muscle activity. Based on these results, the use of a flexible bar just prior to entry into a game is not recommended to enhance power production.

## Figures and Tables

**Table 1:** Experiment 1 force plate results. Comparison of selected maximal jump characteristics following three different HVW protocols. Values expressed as mean ± SD.

HVW Protocol	Jump Height (m)	Avg. Relative Propulsive Force (% BW)	Peak Relative Propulsive Force (% BW)	Time to Takeoff (s)
CON	0.444 ± 0.036	237.04 ± 20.7	299.69 ± 32.1	0.670 ± 0.079
DB	0.443 ± 0.032	236.74 ± 24.3	300.85 ± 36.9	0.679 ± 0.070
FB	0.448 ± 0.029	238.69 ± 22.1	304.98 ± 36.6	0.694 ± 0.093

**Table 2:** Experiment 1 EMG results. Comparison of mean nRMS% electromyographic activity of four muscles following three different HVW protocols. Values expressed as mean ± SD; * significantly different from CON, *p* = 0.13.

HVW Protocol	Mean nRMS% Vastus Lateralis	Mean nRMS% Vastus Medialis Oblique	Mean nRMS% Biceps Femoris	Mean nRMS% Lateral Gastrocnemius
CON	199.01 ± 99.9	369.77 ± 195.7	94.57 ± 51.2	177.86 ± 200.7
DB	182.97 ± 88.5 *	361.87 ± 184.6	116.02 ± 71.3	153.58 ± 69.5
FB	192.14 ± 95.1	423.10 ± 256.9	148.73 ± 135.7	133.42 ± 52.6

**Table 3:** Experiment 2 results. Comparison of mean nRMS% electromyographic activity of four muscles during maximal jumping with dumbbell or flexible bar. Values expressed as mean ± SD.

Instrument Held While Jumping	Mean nRMS% Vastus Lateralis	Mean nRMS% Vastus Medialis Oblique	Mean nRMS% Biceps Femoris	Mean nRMS% Lateral Gastrocnemius
Dumbbell	97.54 ± 41.9	161.3 ± 97.5	63.25 ± 58.9	92.55 ± 47.1
Flexible bar	90.12 ± 35.3	143.56 ± 88.0	53.22 ± 29.6	86.88 ± 38.0

## Data Availability

The data presented in this study are only available upon request from the corresponding author. The data are not publicly available due to privacy issues.
